# Virtual Screening of Small Molecular Inhibitors against DprE1

**DOI:** 10.3390/molecules23030524

**Published:** 2018-02-27

**Authors:** Gang Zhang, Song Guo, Huaqing Cui, Jianguo Qi

**Affiliations:** 1 State Key Laboratory of Bioactive Substances and Function of Natural Medicine, Institute of Materia Medica, Peking Union Medical College and Chinese Academy of Medical Sciences, Beijing 100050, China; hcui@imm.ac.cn; 2 Department of Computer Application, Shenyang Sport University, Shenyang 110102, China; 3 Key Laboratory of Natural Medicine and Immuno-Engineering of Henan Province, Henan University Jinming Campus, Kaifeng 475004, China; qijianguo@henu.edu.cn

**Keywords:** DprE1, virtual screening, tuberculosis, ADMET

## Abstract

Decaprenylphosphoryl-β-d-ribose oxidase (DprE1) is the flavoprotein subunit of decaprenylphosphoryl-d-ribose epimerase involved in cell wall synthesis in *Mycobacterium tuberculosis* and catalyzes the conversion of decaprenylphosphoryl ribose to decaprenylphosphoryl arabinose. DprE1 is a potential target against tuberculosis, including multidrug-resistant tuberculosis. We identified potential DprE1 inhibitors from the ChemDiv dataset through virtual screening based on pharmacophore and molecular docking. Thirty selected compounds were subjected to absorption, distribution, metabolism, excretion, and toxicity prediction with the Discovery Studio software package. Two compounds were obtained as hits for inhibiting DprE1 activity in *M. tuberculosis* and are suitable for further in vitro and in vivo evaluation.

## 1. Introduction

*Mycobacterium tuberculosis* is the causative agent of tuberculosis (TB), a contagious airborne disease of humans that is one of the top 10 causes of death worldwide and has even caused higher mortality than the hunan immunodeficiency virus/acquired immunodeficiency syndrome (HIV/AIDS) since 2015. About one third of the world’s population has latent TB, which means that they have been infected by *M. tuberculosis* bacteria but are not yet ill with TB and cannot transmit the disease. According to the World Health Organization, there were an estimated 10.4 million new TB cases and 1.8 million TB deaths worldwide in 2016. People living with HIV accounted for 1.2 million (11%) of all new TB cases [1]. Due to the emergence of multidrug-resistant TB, extensively drug-resistant (XDR) TB, totally drug-resistant TB, and super-XDR TB [2], there is an urgent need for new drug candidates with new mechanisms of action.

Decaprenylphosphoryl-β-d-ribose oxidase (DprE1) is the flavoprotein subunit of decaprenylphosphoryl-d-ribose epimerase, which is involved in cell wall synthesis and produces decaprenylphosphoryl arabinose (DPA), a unique sugar donor for biogenesis of the essential mycobacterial cell wall polysaccharides arabinogalactan and lipoarabinomannan [3]. DprE1 acts in concert with DprE2 to catalyze the two-step epimerization of decaprenylphosphoryl ribose (DPR) to DPA. DprE1 uses Flavin adenine dinucleotide (FAD) to oxidize DPR to a keto intermediate, which is then reduced to DPA by DprE2 using the reduced form of nicotinamide adenine dinucleotide (NADH) as a cofactor [4,5]. Analysis of orthologs has revealed that DprE1 is essential for the growth of mycobacteria, making it a valuable target for drug development [5].

**Figure 1:**
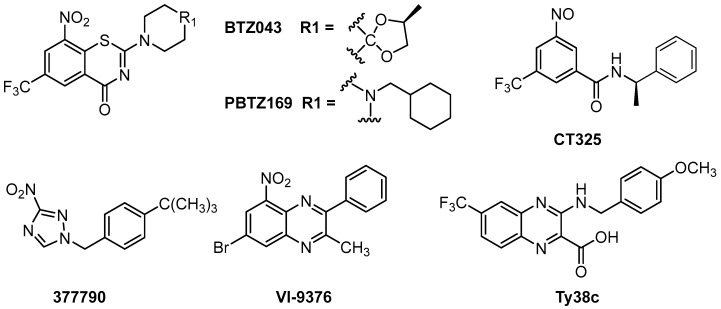
The chemical structures of some Decaprenylphosphoryl-β-d-ribose oxidase (DprE1) inhibitors.

Although some DprE1 inhibitors have been reported, including benzothiazinones, dinitrobenzamides, nitroquinoxalines, and nitroimidazoles (Figure 1), no DprE1 inhibitors are currently in clinical use. Benzothiazinones have been identified as potential candidates for enzyme inhibition, among which BTZ043 and PBTZ169 are the most promising compounds and are currently in clinical trials [6,7]. BTZ043 and PBTZ169 are covalent DprE1 inhibitors, in which the nitro group is reduced to a nitroso group and forms a covalent bond with the thiol group of the active site Cys387 [6,7]. The dinitrobenzene derivative CT325 inhibits DprE1 by a similar mechanism [7]. VI-9376 is the lead among the nitroquinoxalines, which are compounds structurally similar to benzothiazinones [8]. The representative compound of nitroimidazoles, 377790, was also found to bind covalently with Cys 387 in DprE1 [9] Ty38c showed good antitubercular activity as a noncovalent inhibitor of DprE1 [10]. Though no significant disadvantages of these DprE1 inhibitors were reported, there is still much uncertainty preventing any currently known DprE1 inhibitor from being developed as a clinical drug.

Virtual screening for drug discovery is becoming an essential tool in assisting fast, cost-efficient lead discovery and optimization. Rational and structure-based drug design is more efficient than the traditional method of drug discovery because this method examines the molecular basis of a disease and uses the three-dimensional structure of the biological target. In this work, we used virtual screening in silico to identify potential small molecular inhibitors against DprE1.

## 2. Results and Discussion

**Figure 2:**
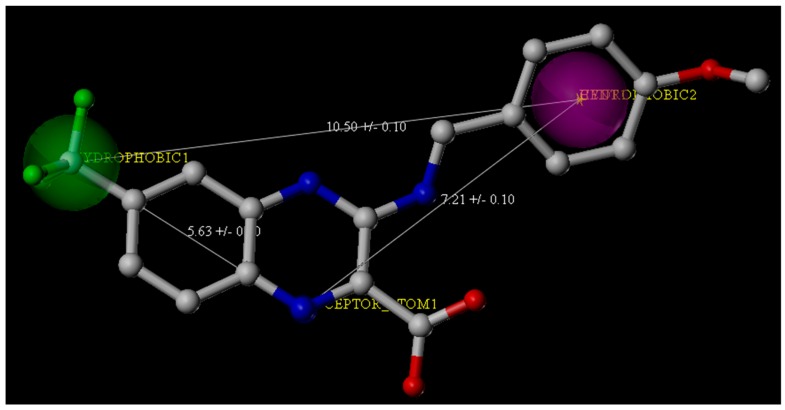
Pharmacophore features derived from the crystal structure of the *M. tuberculosis* DprE1 complex with Ty38c (the Protein Data Bank Code: 4P8K). Ty38c is shown as the stick structure. Green and purple spheres indicate the hydrophobic groups, and the blue spheres indicate the hydrogen bond donors.

The ChemDiv is the industry’s largest, most diverse, and most pharmacologically-relevant commercial collection, containing 1,962,494 individually crafted, lead-like, drug-like small molecules [11]. First, the dataset was filtered using Oprea’s lead-likeness criteria [12]. After analysis of the interaction between DprE1 and Ty38c, the pharmacophore model was established, which consisted of one hydrogen bond donor atom and two hydrophobic features with distance constraints of 5.63 ± 0.1, 7.21 ± 0.1, and 10.5 ± 0.1 Å, respectively (Figure 2). The 941,361 molecules in the filtered database were filtered using three-dimensional (3D) and flexible queries in the parmacophore model generated by the UNITY module of SYBYL-X 2.1. All the conformers of these molecules were generated on the fly during the pharmacophore search.

**Figure 3:**
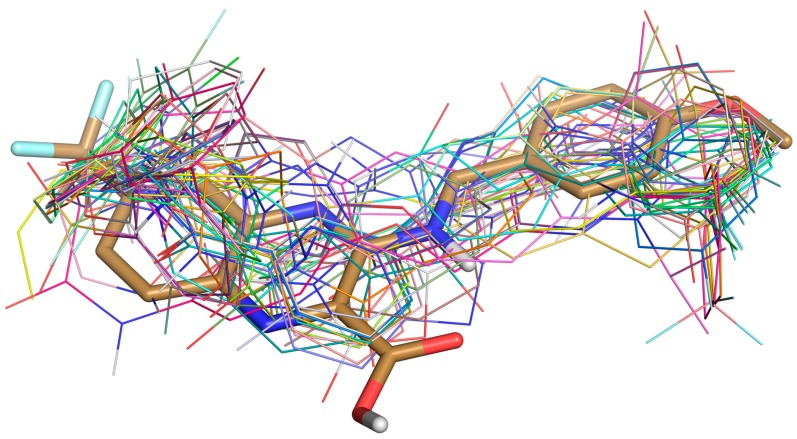
Superposition of Ty38c (in stick) and 30 selected ligands (in lines) docked in DprE1.

A total of 135,755 molecules fitted the pharmacophore features and were subjected to the docking-based virtual screening in Autodock Vina. Thirty molecules were selected to perform the absorption, distribution, metabolism, excretion, and toxicity (ADMET) prediction with the Discovery Studio 2.5 software package (Figure 3). The candidate molecules were selected based on the following considerations. (1) There were no clashes between the ligand and any residues on DprE1; (2) In a cluster of similar molecules, smaller ones were preferred, as they would allow more room for structural optimization; (3) Molecules that formed distinguishable hydrogen bonds (2.5–3.2 Å, 130–180°) and π–π stacking interactions (3–5 Å) with the residues in the binding pocket were preferred.

**Figure 4:**
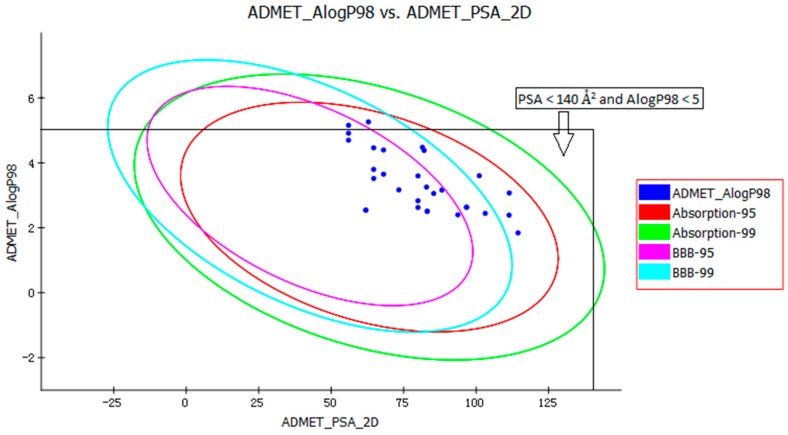
Plot of the two-dimensional polar surface area (PSA_2D) vs. the calculated ALogP98 for tested compounds showing the 95% and 99% confidence limit ellipses corresponding to the blood-brain barrier (BBB) and the human intestinal absorption (HIA) models.

The pharmacokinetic profile of all of the molecules under investigation was predicted by ADMET models provided by the Discovery Studio 2.5 program. The biplot shows the two analogous 95% and 99% confidence ellipses corresponding to the human intestinal absorption (HIA) and the blood-brain barrier (BBB) models. Polar surface area (PSA) had an inverse relationship with percent HIA, and thus with cell wall permeability [13]. Although a relationship between PSA and permeability has been demonstrated, the models usually do not consider the effects of other descriptors. Despite the general use of log P to estimate a compound’s lipophilicity, log P raises concerns about using log P to estimate hydrophilicity and hydrophobicity because it is a ratio [14]. Thus, the hydrogen bonding characteristics obtained by calculating the PSA could be considered along with the log P calculation [15]. Consequently, a model with descriptors Atom-based Log P98 (ALogP98) and two-dimensional PSA (PSA_2D) with a biplot comprising 95% and 99% confidence ellipses was used for the accurate prediction of the compounds’ cell permeability [16]. The 95% confidence ellipse represents the region of chemical space where we can expect to find well-absorbed compounds (≥90%) 95 out of 100 times. The 99% confidence ellipse represents the region of chemical space containing compounds with excellent absorption through cell membranes. According to the model, a compound with optimum cell permeability should follow the criteria PSA < 140 Å^2^ and AlogP98 < 5 [15]. All of the compounds showed PSA < 140 Å^2^, and all compounds, except ZINC23333900 and ZINC64873003, had AlogP98 < 5 (Figure 4).

According to the ADMET prediction, six of the 30 compounds possessed high penetration capacity and 10 compounds possessed medium penetration capacity. Four of the 30 compounds were outside the 99% BBB confidence ellipse, which meant that the quality of the results was unknowable (undefined level of 4). All 30 compounds were inside the 99% absorption ellipse, so they were expected to possess good HIA. The solubility of tested compounds varied from very low to good. The aqueous solubility of five of the 30 compounds was good (Table 1). Cytochrome P450 2D6 (CYP2D6) is involved in the metabolism of a wide range of xenobiotics and its inhibition by a drug may lead to serious drug-drug interactions [17]. Therefore, determining CYP2D6 inhibition is a vital part of the drug discovery and development process. The compounds, except ZINC04724734, were classified as non-inhibitors of CYP2D6. The hepatotoxicity model predicts the incidence of dose-dependent human toxicity [18]. According to the Discovery Studio 2.5 Hepatotoxicity model, 17 of the 30 compounds were classified as non-hepatotoxic. The pharmaceutical activity is determined by the free drug concentration; therefore, the possible plasma protein binding of compounds must be considered [19]. Six of the 30 tested compounds were likely to be <90% binding, five were likely to be ≥90% binding, and 19 were likely to be ≥95% binding (Table 1).

Due to the ADMET predictions, the docking studies, and chemical structures (refers to structural modification), ZINC09833455 and ZINC32996629 are suitable for further in vitro and in vivo evaluation.

**Figure 5:**
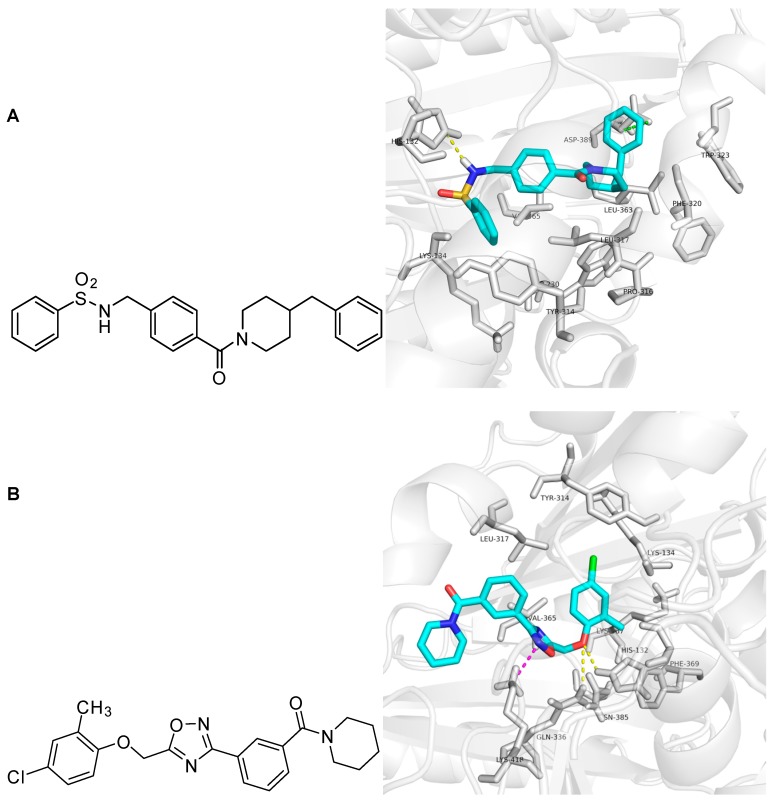
Docking study of ZINC09833455 and ZINC32996629 in DprE1 shown by Pymol 0.99. Panel (**A**): Chemical structure and molecular modeling of ZINC09833455. ZINC09833455 is shown as the cyan stick structure and the surrounding residues are gray. Hydrogen bond interactions are yellow dotted lines and anion-π interactions are green dotted lines; Panel (**B**): Chemical structure and molecular modeling of ZINC32996629. ZINC32996629 is shown as the cyan stick structure and the surrounding residues are gray. Hydrogen bond interactions are yellow dotted lines and cation-π interactions are purple dotted lines.

In the docking study, ZINC09833455 formed hydrogen bond interactions with N of the His 132 residue. The COO- group of Asp 389 showed anion-π interactions with the phenyl ring of ZINC09833455, which was surrounded by the hydrophobic groups of Trp230, Pro316, Phe320, Trp323, and Leu363. The middle phenyl ring of ZINC09833455 showed hydrophobic interactions with the corresponding parts of Lys134, Tyr314, Leu317, and Val365. ZINC32996629 formed hydrogen bonds with His132 and Gln336. The chloro-substituted phenyl ring occupied the hydrophobic site formed by Lys134, Tyr314, Leu317, Val365, Lys367, and Phe369. The cation-π interaction between Lys418 and the 1,2,4-oxadiazole ring of ZINC32996629 is shown in Figure 5. Thus, ZINC09833455 and ZINC32996629 could be used as potential hits for further in vitro and in vivo evaluation.

## 3. Materials and Methods

### 3.1. Preparation for Virtual Screening

The ChemDiv dataset containing 1.96 million compounds was downloaded from the ZINC database [11]. The Protein Drug Bank (PDB) structure of DprE1 protein (PDB Code: 4P8K) was retrieved from the PDB (http://www.rcsb.org/pdb/home/home.do).

### 3.2. First Round of Screening Based on Oprea’s Lead-Likeness Criteria

The ChemDiv dataset, containing 1,962,494 compounds, was filtered to exclude compounds that did not follow Oprea’s lead-likeness criteria [12] (150 < MW < 450, −3.5 < C log P < 4.5, 0 < number of rings < 4, 0 < rotational bonds < 10, 0 < donors < 5, 0 < acceptors < 8). The filtered database contained 941,361 compounds.

### 3.3. Second Round of Screening Based on Pharmacophore Models

We composed queries based on pharmacophores identified from the crystal structure of PDB Code 4P8K [10]. Based on the analysis of the interactions within the binding pocket, the UNITY module in SYBYL-X 2.1 was used for the virtual screening by default (at least four out of three features and three constraints in the pharmacophore model had to be matched). From the second round virtual screening, 135,755 compounds were obtained. The pharmacophore features for the second round of screening were two hydrophobic groups and one hydrogen bond donor with three distance constraints (5.63 ± 0.1, 7.21 ± 0.1, and 10.5 ± 0.1 Å) (Figure 2).

### 3.4. Third Round of Screening Based on Docking

AutoDock Vina (Scripps Research Institute, San Diego, CA, USA) was used to screen 135,755 compounds in the database by docking [20]. For the docking studies, the X-ray crystallographic structure of the DprE1 complex with Ty38c (PDB Code: 4P8K) from the Protein Data Bank (PDB) was used. The PDB protein and molecules were prepared by adding hydrogen and missing residues in Sybyl-X 2.1 and converted to pdbqt format by Openbabel [21]. A grid box with dimensions of 30 × 30 × 30 Å (38.972, 12.580, 10.750) with a spacing of 0.375 Å was constructed around the docking area using Autogrid 4.2 software [22]. Molecules were docked using Vina with exhaustiveness grade 8, with up to nine poses saved per molecule. The docking procedure was carried out for the unchanged conformation of the receptor and flexible ligand molecules. Ty38c was redocked in the 4P8K model to validate the docking algorithms of AutoDock Vina (the root-mean-square deviation value is 0.844). The lowest energy conformations were selected and the ligand interactions with DprE1 were determined. Accelrys Discovery Studio Visualizer 4.0 (Accelrys, San Diego, CA, USA) was used for interaction visualization. Through inspection of the top docking poses, 30 compounds were selected from the top 200 compounds based on the binding affinity (Table S1).

### 3.5. ADMET Prediction

To estimate the drug-likeness of the compounds, in silico absorption, distribution, metabolism, excretion, and toxicity (ADMET) prediction was carried out [23]. We investigated the ADMET properties of the 30 selected compounds using the ADMET Protocol in the Discovery Studio 2.5 software package (Accelrys, San Diego, CA, USA). These studies were solely based on the chemical structure of the molecule. Some of the parameters that were calculated included Atom-based Log P98 (ALogP98), ADME 2D Fast Polar Surface Area (ADME 2D FPSA), Blood Brain Barrier (BBB), Cytochrome P4502D6 (CYP2D6), and Hepatotoxicity (HEPATOX).

## 4. Conclusions

DprE1 is the flavoprotein subunit of decaprenylphosphoryl-d-ribose epimerase involved in cell wall synthesis in *M. tuberculosis* and catalyzes the conversion of DPR to DPA. We identified DprE1 inhibitors from the ChemDiv dataset through virtual screening based on pharmacophore and molecular docking. ADMET prediction was performed on 30 selected compounds with the Discovery Studio software package. Compounds ZINC09833455 and ZINC32996629 were obtained as hits for inhibiting the DprE1 activity of TB and may be suitable for further in vitro and in vivo evaluation.

## Figures and Tables

**Table 1:** The absorption, distribution, metabolism, excretion, and toxicity (ADMET) predictions for 30 selected compounds.

Compounds	BBB Level ^a^	Absorption Level ^b^	Solubility Level ^c^	Hepatotoxicity ^d^	CYP2D6 ^e^	PPB Level ^f^	AlogP98	PSA 2D
ZINC04497363	3	0	2	0	0	2	2.506	83.12
ZINC04497364	3	0	2	0	0	2	2.506	83.12
ZINC04724734	2	0	2	1	1	1	4.478	81.485
ZINC05196271	2	0	2	1	0	2	3.253	82.903
ZINC08614786	2	0	2	1	0	2	3.167	73.348
ZINC09502318	3	0	2	0	0	2	3.16	88.19
ZINC09743860	2	0	2	1	0	1	4.382	82.052
ZINC09833455	1	0	2	0	0	2	4.395	68.065
ZINC12249931	4	0	2	1	0	0	3.602	101.082
ZINC15858572	3	0	2	1	0	0	3.056	85.365
ZINC15865571	2	0	2	1	0	2	3.597	79.947
ZINC17149731	2	0	2	0	0	1	3.65	68.065
ZINC21100102	3	0	3	1	0	2	2.624	79.947
ZINC21100117	3	0	3	1	0	2	2.83	79.947
ZINC21884825	3	0	3	0	0	2	2.444	103.141
ZINC21884833	2	0	2	0	0	2	3.797	64.659
ZINC23333900	1	0	1	1	0	2	5.261	62.856
ZINC32996629	1	0	2	0	0	2	4.462	64.659
ZINC33123019	4	0	2	0	0	0	3.071	111.432
ZINC33257862	2	0	2	0	0	2	3.517	64.659
ZINC35480955	1	0	2	0	0	1	4.912	55.985
ZINC35511826	1	0	2	0	0	1	4.696	55.985
ZINC36159960	2	0	2	0	0	0	2.541	61.959
ZINC36159963	2	0	2	0	0	0	2.541	61.959
ZINC49421663	4	0	2	0	0	2	2.389	111.364
ZINC49421722	3	0	2	1	0	2	2.632	96.689
ZINC49421723	3	0	2	1	0	2	2.632	96.689
ZINC64873003	1	0	2	0	0	2	5.154	55.985
ZINC64890370	4	0	3	1	0	2	1.843	114.464
ZINC65318827	3	0	3	0	0	0	2.399	93.683

^a^ 0, 1, 2, 3, and 4 denote very high, high, medium, low, and undefined, respectively. ^b^ 0, 1, 2, and 3 denote good absorption, moderate absorption, low absorption, and very low absorption, respectively. ^c^ 0, 1, 2, 3, 4, and 5 denote extremely low, very low but possible, low, good, optimal, and too soluble, respectively. ^d^ 0 and 1 represent nontoxic and toxic, respectively. ^e^ 0 and 1 denote noninhibitor and inhibitor, respectively. ^f^ 0, 1, and 2 indicate <90% binding, ≥90% binding, and ≥95% binding, respectively.

## Supplementary Materials

The following are available online, Table S1. Top 200 compounds and their binding affinity.

## References

[1] (2016). Global Tuberculosis Report 2016.

[2] Velayati A.A., Masjedi M.R., Farnia P., Tabarsi P., Ghanavi J., ZiaZarifi A.H., Hoffner S.E. (2009). Emergence of new forms of totally drug-resistant tuberculosis bacilli. Chest.

[3] Wolucka B.A. (2008). Biosynthesis of d-Arabinose in mycobacteria—A novel bacterial pathway with implications for antimycobacterial therapy. FEBS J..

[4] Mikusova K., Huang H., Yagi T., Holsters M., Vereecke D., D’Haeze W., Scherman M.S., Brennan P.J., McNeil M.R., Crick D.C. (2005). Decaprenylphosphoryl arabinofuranose, the donor of the d-arabinofuranosyl residues of mycobacterial arabinan, is formed via a two-step epimerization of decaprenylphosphoryl ribose. J. Bacteriol..

[5] Makarov V., Manina G., Mikusova K., Möllmann U., Ryabova O., Saint-Joanis B., Dhar N., Pasca M.R., Buroni S., Lucarelli A.P. (2009). Benzothiazinones kill Mycobacterium tuberculosis by blocking arabinan synthesis. Science.

[6] Makarov V., Lechartier B., Zhang M., Neres J., van der Sar A.M.A.M., Raadsen S.A.S.A., Hartkoorn R.C.R.C., Ryabova O.B.O.B., Vocat A., Decosterd L.A.L.A. (2014). Towards a new combination therapy for tuberculosis with next generation benzothiazinones. EMBO Mol. Med..

[7] Trefzer C., Rengifo-Gonzalez M., Hinner M.J., Schneider P., Makarov V., Cole S.T., Johnsson K. (2010). Benzothiazinones: Prodrugs that covalently modify the decaprenylphosphoryl-β-d-ribose 2′-epimerase DprE1 of mycobacterium tuberculosis. J. Am. Chem. Soc..

[8] Magnet S., Hartkoorn R.C., Székely R., Pató J., Triccas J.A., Schneider P., Szántai-Kis C., Őrfi L., Chambon M., Banfi D. (2010). Leads for antitubercular compounds from kinase inhibitor library screens. Tuberculosis.

[9] Stanley S.A., Grant S.S., Kawate T., Iwase N., Shimizu M., Wivagg C., Silvis M., Kazyanskaya E., Aquadro J., Golas A. (2012). Identification of novel inhibitors of m. tuberculosis growth using whole cell based high-throughput screening. ACS Chem. Biol..

[10] Neres J., Hartkoorn R.C., Chiarelli L.R., Gadupudi R., Pasca M.R., Mori G., Venturelli A., Sacina S., Makarov V., Kolly G.S. (2015). 2-Carboxyquinoxalines kill Mycobacterium tuberculosis through noncovalent inhibition of DprE1. ACS Chem. Biol..

[11] ChemDiv Dataset ZINC Database. http//zinc.docking.org/catalogs/cdiv.

[12] Oprea T.I., Davis A.M., Teague S.J., Leeson P.D. (2001). Is there a difference between leads and drugs? A historical perspective. J. Chem. Inf. Comput. Sci..

[13] Palm K., Stenberg P., Luthman K., Artursson P. (1997). Polar molecular surface properties predict the intestinal absorption of drugs in humans. Pharm. Res..

[14] Leo A., Hansch C., Elkins D. (1971). Partition coefficients and their uses. Chem. Rev..

[15] Egan W.J., Merz K.M., Baldwin J.J. (2000). Prediction of drug absorption using multivariate statistics. J. Med. Chem..

[16] (2011). Chemistry Collection: Basic Chemistry User Guide, Pipeline Pilot.

[17] Wang B., Yang L.-P., Zhang X.-Z., Huang S.-Q., Bartlam M., Zhou S.-F. (2009). New insights into the structural characteristics and functional relevance of the human cytochrome P450 2D6 enzyme. Drug Metab. Rev..

[18] Pirmohamed M., Breckenridge A.M., Kitteringham N.R., Park B.K. (1998). Fortnightly review: Adverse drug reactions. BMJ Br. Med. J..

[19] Moroy G., Martiny V.Y., Vayer P., Villoutreix B.O., Miteva M.A. (2012). Toward in silico structure-based ADMET prediction in drug discovery. Drug Discov. Today.

[20] Trott O., Olson A.J. (2010). AutoDock Vina: Improving the speed and accuracy of docking with a new scoring function, efficient optimization, and multithreading. J. Comput. Chem..

[21] O’Boyle N.M., Banck M., James C.A., Morley C., Vandermeersch T., Hutchison G.R. (2011). Open Babel: An open chemical toolbox. J. Cheminform..

[22] Autogrid. http://autodock.scripps.edu/wiki/AutoGrid.

[23] Lagorce D., Reynes C., Camproux A.-C., Miteva M.A., Sperandio O., Villoutreix B.O. (2010). In silico adme/tox Predictions. ADMET for Medicinal Chemists.

