# Supplementary material for: Virtual Screening of Small Molecular Inhibitors against DprE1
Source: Molecules. 2018 Feb 27;23(3):524. doi: 10.3390/molecules23030524 (PMC6017230; doi:10.3390/molecules23030524)
Supplement: Supplementary file 1 [file molecules-23-00524-s001.pdf]

Table S1. Top 200 compounds and their binding affinity

Cmpd	Binding affinity (Kcal/mol)
Ty38c	-7.1
ZINC04724734	-12.7
ZINC23333900	-12.6
ZINC65318827	-12.6
ZINC15865571	-12.5
ZINC21884825	-12.5
ZINC12249931	-12.4
ZINC21100117	-12.4
ZINC32996629	-12.4
ZINC35511826	-12.4
ZINC36159960	-12.4
ZINC36159963	-12.4
ZINC04497363	-12.3
ZINC04497364	-12.3
ZINC05196271	-12.3
ZINC08614786	-12.3
ZINC09502318	-12.3
ZINC09743860	-12.3
ZINC15858572	-12.3
ZINC21884833	-12.3
ZINC35480955	-12.3
ZINC64890370	-12.3
ZINC09833455	-12.2
ZINC17149731	-12.2
ZINC21100102	-12.2
ZINC33123019	-12.2
ZINC33257862	-12.2
ZINC49421663	-12.2
ZINC49421722	-12.2
ZINC49421723	-12.2
ZINC64873003	-12.2
ZINC00692536	-12.1
ZINC00826777	-12.1
ZINC01427162	-12.1
ZINC02107854	-12.1
ZINC02107856	-12.1
ZINC02260318	-12.1
ZINC03020783	-12.1
ZINC03020785	-12.1

ZINC03020787	-12.1
ZINC05091990	-12.1
ZINC09152241	-12.1
ZINC15776169	-12.1
ZINC17200320	-12.1
ZINC21530224	-12.1
ZINC33013733	-12.1
ZINC35476678	-12.1
ZINC35476679	-12.1
ZINC49421709	-12.1
ZINC61718743	-12.1
ZINC64747147	-12.1
ZINC64930322	-12.1
ZINC64930323	-12.1
ZINC64960571	-12.1
ZINC65195228	-12.1
ZINC65197681	-12.1
ZINC65312712	-12.1
ZINC65312713	-12.1
ZINC65318796	-12.1
ZINC96320636	-12.1
ZINC96321504	-12.1
ZINC96397371	-12.1
ZINC97112853	-12.1
ZINC00797604	-12.0
ZINC01124636	-12.0
ZINC02841731	-12.0
ZINC04474735	-12.0
ZINC04494378	-12.0
ZINC05033175	-12.0
ZINC06751947	-12.0
ZINC06819072	-12.0
ZINC07755725	-12.0
ZINC08592343	-12.0
ZINC09743768	-12.0
ZINC15418694	-12.0
ZINC15780192	-12.0
ZINC17178146	-12.0
ZINC20484556	-12.0
ZINC20990127	-12.0
ZINC21100107	-12.0
ZINC21884731	-12.0
ZINC21884733	-12.0

ZINC33067841	-12.0
ZINC33261729	-12.0
ZINC33268589	-12.0
ZINC33268590	-12.0
ZINC33276548	-12.0
ZINC49006292	-12.0
ZINC64890790	-12.0
ZINC64943512	-12.0
ZINC65318820	-12.0
ZINC65318823	-12.0
ZINC65318832	-12.0
ZINC75437537	-12.0
ZINC96307756	-12.0
ZINC96324169	-12.0
ZINC00710936	-11.9
ZINC00797605	-11.9
ZINC01499239	-11.9
ZINC02355880	-11.9
ZINC02959814	-11.9
ZINC02972975	-11.9
ZINC03014128	-11.9
ZINC03024992	-11.9
ZINC03324267	-11.9
ZINC03550677	-11.9
ZINC04496949	-11.9
ZINC04743987	-11.9
ZINC05042555	-11.9
ZINC05053329	-11.9
ZINC05053360	-11.9
ZINC05136868	-11.9
ZINC05399764	-11.9
ZINC05450631	-11.9
ZINC06738644	-11.9
ZINC06751946	-11.9
ZINC06940744	-11.9
ZINC08598244	-11.9
ZINC08600467	-11.9
ZINC08607165	-11.9
ZINC09743704	-11.9
ZINC09743914	-11.9
ZINC09830882	-11.9
ZINC09830883	-11.9
ZINC12006142	-11.9

ZINC15682057	-11.9
ZINC15784632	-11.9
ZINC15826681	-11.9
ZINC15858668	-11.9
ZINC15882440	-11.9
ZINC17200322	-11.9
ZINC20062072	-11.9
ZINC20492622	-11.9
ZINC20562634	-11.9
ZINC20934351	-11.9
ZINC20934357	-11.9
ZINC21884743	-11.9
ZINC27528231	-11.9
ZINC32932076	-11.9
ZINC33069096	-11.9
ZINC33131325	-11.9
ZINC33131387	-11.9
ZINC33300723	-11.9
ZINC35373405	-11.9
ZINC65195229	-11.9
ZINC65210627	-11.9
ZINC65210793	-11.9
ZINC65318829	-11.9
ZINC65318889	-11.9
ZINC65318891	-11.9
ZINC65318927	-11.9
ZINC65319009	-11.9
ZINC75437577	-11.9
ZINC75437580	-11.9
ZINC96307774	-11.9
ZINC96320596	-11.9
ZINC96320616	-11.9
ZINC96397668	-11.9
ZINC00797609	-11.8
ZINC01124638	-11.8
ZINC01187686	-11.8
ZINC01461792	-11.8
ZINC02095973	-11.8
ZINC02459997	-11.8
ZINC02460003	-11.8
ZINC02465099	-11.8
ZINC03023998	-11.8
ZINC03061676	-11.8

ZINC04157046	-11.8
ZINC05139321	-11.8
ZINC05268831	-11.8
ZINC06260722	-11.8
ZINC06771869	-11.8
ZINC06772823	-11.8
ZINC06818944	-11.8
ZINC06818945	-11.8
ZINC06819070	-11.8
ZINC08831416	-11.8
ZINC09276372	-11.8
ZINC09408038	-11.8
ZINC09408039	-11.8
ZINC11867488	-11.8
ZINC12862979	-11.8
ZINC13131479	-11.8
ZINC13131481	-11.8
ZINC13131483	-11.8
ZINC13681187	-11.8
ZINC15682054	-11.8
ZINC15776163	-11.8
ZINC15776311	-11.8
ZINC15883194	-11.8
ZINC16955057	-11.8
ZINC17052718	-11.8
ZINC17052720	-11.8
ZINC20285897	-11.8
ZINC20516824	-11.8
ZINC20574391	-11.8
ZINC20934332	-11.8
ZINC20934470	-11.8
ZINC13521724	-11.8
ZINC20557942	-11.8
